# Gait speed and cognitive performance in older adult women: the Study of Women’s Health Across the Nation

**DOI:** 10.1093/geroni/igaf122.117

**Published:** 2025-12-31

**Authors:** Jillian Baker, Lauren MacConnachie, Alexis Reeves, Michelle Hood, Kelley Pettee Gabriel, Bradley Appelhans, Carol Derby, Carrie Karvonen-Gutierrez

**Affiliations:** University of Michigan School of Public Health, Ann Arbor, Michigan, United States; University of Michigan, Ann Arbor, Michigan, United States; Stanford Medicine - Stanford University, Stanford, California, United States; University of Michigan School of Public Health, Ann Arbor, Michigan, United States; The University of Alabama at Birmingham, Birmingham, Alabama, United States; Rush University, Chicago, Illinois, United States; Albert Einstein College of Medicine, Bronx, New York, United States; University of Michigan, Ann Arbor, Michigan, United States

## Abstract

Cross-sectional studies show a bi-directional association between gait speed and cognitive function; longitudinal studies are needed to determine the true causal directionality. However, longitudinal studies, particularly of older adults, are prone to selection bias due to differential loss to follow-up. This analysis leverages longitudinal data from the Study of Women’s Health Across the Nation to explore relationships between gait speed and cognitive function, accounting for meaningful loss-to-follow-up. We hypothesize that gait speed predicts cognitive functioning and vice-versa. SWAN is a multi-ethnic, community-based cohort of women followed through menopause and into older adulthood. At study visits in 2012-13 (V13) and 2015-16 (V15), 1,258 participants completed: a four-meter timed walk to quantify gait speed and tests of working memory, processing speed, and verbal memory. Linear regressions predicted V15 cognitive test scores from V13 gait speed and vice-versa. Inverse probability weights for missingness due to death, stroke, poor physical function, and other missingness adjusted for differential loss-to-follow-up from baseline. In models including weights to account for differential loss-to-follow-up and adjusting for race and ethnicity, education, age, and financial strain, faster gait speeds at V13 were associated with better processing speed at V15 (β = 4.19,95%CI:1.69,6.70) but processing speed at V13 was not statistically significantly associated with gait speed at V15 (β = 0.0010,95%CI:-0.0002,0.0022). No statistically significant associations were observed between gait speed and working or verbal memory. These results suggest the relationship between gait speed and cognitive function is not bi-directional, but that gait speed may have an effect on future processing speed and not the reverse.

